# The Pleiotropic Effects of Vitamin D in Gynaecological and Obstetric Diseases: An Overview on a Hot Topic

**DOI:** 10.1155/2015/986281

**Published:** 2015-04-27

**Authors:** Francesca Colonese, Antonio Simone Laganà, Elisabetta Colonese, Vincenza Sofo, Francesca Maria Salmeri, Roberta Granese, Onofrio Triolo

**Affiliations:** ^1^Department of Oncology, San Raffaele Scientific Institute, 20132 Milan, Italy; ^2^Department of Pediatric, Gynaecological, Microbiological and Biomedical Sciences, University of Messina, 98125 Messina, Italy; ^3^Department of Obstetrics and Gynecology, Macedonio Melloni Hospital, University of Milan, 20129 Milan, Italy; ^4^Department of Environmental Sciences, Safety, Territory, Food and Health, University of Messina, 98125 Messina, Italy

## Abstract

The traditionally recognized role of vitamin D consists in the regulation of bone metabolism and calcium-phosphorus homeostasis but recently a lot of in vitro and in vivo studies recognized several “noncalcemic” effects of vitamin D metabolites. Accumulating evidence suggests that the metabolic pathways of this vitamin may play a key role in the developing of gynaecological/obstetric diseases. VDR-mediated signalling pathways and vitamin D levels seem to (deeply) affect the risk of several gynaecological diseases, such as polycystic ovary syndrome (PCOS), endometriosis, and ovarian and even breast cancer. On the other hand, since also the maternal-fetal unit is under the influence of vitamin D, a breakdown in its homeostasis may underlie infertility, preeclampsia, and gestational diabetes mellitus (GDM). According to our literature review, the relationship between vitamin D and gynaecological/obstetric diseases must be replicated in future studies which could clarify the molecular machineries behind their development. We suggest that further investigation should take into account the different serum levels of this vitamin, the several actions which arise from the binding between it and its receptor (taking into account its possible polymorphism), and finally the interplay between vitamin D metabolism and other hormonal and metabolic pathways.

## 1. Introduction

The traditionally recognized role of vitamin D consists in the regulation of bone metabolism and calcium-phosphorus homeostasis but recently a lot of in vitro and in vivo studies recognized several “noncalcemic” effects of vitamin D metabolites [1]. Reduced levels of vitamin D are linked with the onset and progression of various diseases such as autoimmune diseases including diabetes mellitus type 1, respiratory infections, type 2 diabetes, hypertension and cardiovascular disease [2], neuromuscular disorders, and cancer [3]. Sunlight exposure is the primary source of vitamin D. The synthesis of vitamin D starts in the bowel epithelial with the oxidation of cholesterol from food or bile to pro-vitamin D3 (7-dehydrocholesterol), which is then transported to the skin, mainly the epidermis, wherein it is isomerized to pre-vitamin D3 (cholecalciferol) by UVB radiation. It is then metabolized into two different substances within the body: 25(OH)D3 or calcidiol and 1,25(OH)2D3 or calcitriol. Vitamin D can also be taken from the diet. Decreased sun exposure limits vitamin D synthesis. There are two principal enzymes involved in the formation of circulating 1,25(OH)2D3 from dietary absorbed or skin synthesized vitamin D: the hepatic microsomal or mitochondrial vitamin D 25-hydroxylase (CYP27A1) and the renal mitochondrial enzyme 1*α*-hydroxylase (CYP27B1) for vitamin D and 25(OH)D3, respectively [4, 5]. These hydroxylases belong to a class of proteins known as cytochrome P450 mixed function monooxidases. Extrarenal activity of 25(OH)D3-1*α*-hydroxylase (CYP27B1) has been reported in various cell types including macrophages, keratinocytes, prostate, and colon cancer cells [6–8]. It was shown that 1,25(OH)2D3 is produced locally in many tissues. The potent fat soluble seco-steroid hormone 1,25(OH)2D3 acts via binding to a corresponding nuclear receptor called the “vitamin D receptor” (VDR) [9–11]. The VDR represents the final common pathway through which vitamin D works on target tissues. The VDR is widely distributed across many tissues. This widespread distribution underlies the potential myriad of physiologic actions for vitamin D. 1,25(OH)2D are mediated by the VDR acting primarily by regulating the expression of genes whose promoters contain specific DNA sequences known as vitamin D response elements (VDRE). The VDR works in partnership with other transcriptional factors, the best-studied of which is the retinoid X receptor (RXR), and a number of coactivators and corepressors that provide context, tissue, and target gene specificity. However, some actions of 1,25(OH)2D are faster than genomic and may be mediated by a membrane-bound VDR that has been less well characterized than the nuclear VDR [12]. VDR belongs to the superfamily (>150 members) of transacting transcriptional regulatory factors, which includes the steroid and thyroid hormone receptors [13, 14] and is encoded by a large gene (>100 kb) located on the chromosome 12q12-14 [15]. The VDR gene encompasses two promoter regions, eight protein-coding exons (namely, 2–9), and six untranslated exons (1a–1f). It has an extensive promoter region capable of generating multiple tissue-specific transcripts. It has been demonstrated that VDR requires heterodimerization with auxiliary proteins for effective DNA interaction. These auxiliary proteins have been identified as the retinoid-X receptors (RXR) *α*, s, and *γ* [13, 14, 16, 17]. Vitamin D response elements have been identified in numerous genes involved in many activities (i.e., cellular growth, differentiation, apoptosis, invasion and metastasis of tumour cells, and so on).

Considering these assumptions, in this paper we aimed to review the most updated evidence which clearly suggests a key role for vitamin D pleiotropic actions in the reproductive physiology as well as development of several gynecologic/obstetric diseases. In particular, we discuss the influence of VDR-mediated signaling pathways in polycystic ovary syndrome (PCOS), gestational diabetes mellitus (GDM), preeclampsia, infertility and in vitro fertilization (IVF), endometriosis, and breast and ovarian cancer.

## 2. Vitamin D, Polycystic Ovary Syndrome, and Insulin Resistance

PCOS is the most common gynaecological endocrinopathy in women of reproductive age, with a prevalence of 6–10% in the general population. It is a multigenic disorder characterized by increased ovarian and adrenal androgen secretion; hyperandrogenic symptoms such as hirsutism, acne, and/or alopecia; menstrual irregularity; and polycystic ovaries [18–21]. In addition, insulin resistance (IR) is common in PCOS women [22] who are therefore at an increased risk of type 2 diabetes [23]. There is an increasing evidence that supports the contribution of vitamin D deficiency to metabolic disturbances in women with PCOS, including insulin resistance (IR) [24–26], obesity [24, 27], hypertension [28], and menstrual dysfunction [29], findings supported by the fact that vitamin D regulates about 3% of the human genome, including genes that are crucial for glucose and lipid metabolism [30, 31]. Consistent evidence also suggests that polymorphisms in the VDR gene are associated with vitamin D deficiency in PCOS and its metabolic and endocrine disturbances [26, 32]. The VDR Cdx2 “AA” genotype is reported as an associated marker with lower fasting insulin and homeostatic model assessment-IR [26] and the ApaI “CC” genotype was associated with an increased risk for PCOS [32]. The exact mechanisms underlying the association of vitamin D and IR are not fully understood. Firstly, vitamin D may have a beneficial effect on insulin action by stimulating the expression of insulin receptor and thereby enhancing insulin responsiveness for glucose transport [33]. The vitamin D responsive element is present in the promoter of the human insulin gene [34] and the transcription of the human insulin gene is activated by 1,25(OH)D2 [35]. Secondly, vitamin D regulates extracellular and intracellular calcium that is essential for insulin-mediated intracellular processes in insulin-responsive tissues such as skeletal muscle and adipose tissue [33]. Moreover, alterations in calcium flux can have adverse effects on insulin secretion, which is a calcium dependent process [36]. Finally, as vitamin D has a modulating effect on the immune system [37], hypovitaminosis D might induce a higher inflammatory response, which is again associated with IR [38]. Literature assessing VDR polymorphisms and/or polymorphisms related to vitamin D metabolism in women suffering from PCOS in relation to vitamin D status and metabolic disturbances is scarce. Recently, Krul-Poel et al. [39] have carried out a review on this topic. They found 29 eligible trials with inconsistency in their results. The conflicting findings might be due to the small sample sizes, the lack of adjustments for confounders, the use of different definitions for PCOS, the use of different assays for serum 25(OH)D measurement, the duration of intervention, the use of different amounts of vitamin D supplementation in the intervention trials, and the lack of an optimal serum 25(OH)D level in the general population. They underline that only one well-designed randomized placebo-controlled trial demonstrating no effect of vitamin D3 supplementation on IR has been carried out until now [40]. Moreover, they found that univariate regression analyses of the weighted means revealed vitamin D to be a significant and independent predictor of IR in both PCOS and control women. The significance disappeared after adjustment for BMI in PCOS women [39]. Still, it remains unclear whether vitamin D and IR are causally interrelated or whether they constitute two independent characteristics in women with PCOS. The causal relationship between vitamin D status and metabolic disturbances in PCOS remains to be determined in well-designed placebo-controlled randomized clinical trials. Until then, screening women who are at risk of vitamin D deficiency and supplementation with vitamin D could be considered.

## 3. Vitamin D and Gestational Diabetes Mellitus

GDM is a condition of abnormal maternal glucose tolerance that occurs, or is detected for the first time, during pregnancy [41]. Pregnancy is a status in which the mother undergoes physiological insulin resistance, which helps the fetus absorb more nutrients. Maternal postprandial hyperglycaemia is the reason why the fetus can take in more carbohydrates and amino acids via the placenta, thanks to a carrier passage, the functioning of which is facilitated by the different gradient (typically facilitated transport). If the mother is unable to compensate with an increase of pancreatic *β*-cell insulin secretion, GDM is derived from this metabolic condition [42, 43]. Women affected by GDM generally demonstrate in the puerperium, and/or later in life, a maintenance of high levels of insulin resistance, which is the effect of *β*-cell dysfunction, and suggests that GDM is a transient manifestation of longstanding metabolic impairment with a predisposition to reappear in the future [44]. There is a strict connection between glucose metabolism and vitamin D pathways [45]: it is widely accepted, for example, that this vitamin and PTH play a key role in the extracellular homeostasis of calcium [46], and moreover that patients affected by hyperparathyroidism develop more frequently diabetes mellitus type 2 with respect to the general population [47, 48]. Since 1,25(OH)2D is able to induce insulin secretion and to decrease insulin resistance, low levels of this vitamin are associated with the developing of GDM. A cross-sectional study conducted by Maghbooli et al. [49] on 741 pregnant women showed that prevalence of severe vitamin D deficiency (<12.5 nmol/L; <5 ng/mL) in GDM patients was higher than in normoglycaemic pregnancies and found a strong correlation between the HOMA index and serum levels of vitamin D. Confirming these results, Zhang et al. [50] found that approximately 33% of GDM cases in their study population, compared with 14% of controls (*P* < 0.001), had maternal plasma 25(OH)D concentrations consistent with a prespecified diagnosis of vitamin D deficiency (<50 nmol/L; <20 ng/mL). Moreover, each 12,5 nmol/L (5 ng/mL) decrease in 25(OH)D concentrations was related to a 1.29-fold increase in GDM risk. Finally, Zuhur et al. [51] evidenced that correlation between low levels of vitamin D and risk of GDM still remain even after adjusting for well-established risk factors (maternal age, race, family history of diabetes, and pre-pregnancy BMI) of GDM. Nevertheless, another study [52] did not find evidence of an association between first-trimester maternal levels of 25(OH)D and subsequent development of GDM. Taken together, all these results allow us to underline the strict correlation between vitamin D and glucose metabolisms, even if further studies based on larger population are needed to get crystal clear evidence about the topic.

## 4. Vitamin D and Preeclampsia

Impaired placentation and maternal endothelial dysfunction are principal features of the pregnancy syndrome preeclampsia that affects 3–7% of all pregnancies [53, 54]. Effective preventive or therapeutic strategies do not exist to date [53]. Vitamin D3 deficiency is associated with cardiovascular disease, hypertension, obesity, diabetes mellitus, and metabolic syndrome [54, 55]. Although the mechanisms through which low serum vitamin D levels can affect the risk of preeclampsia are still unclear, the causal relationship is biologically plausible. Vitamin D has been shown as a potent endocrine suppressor of renin biosynthesis to regulate the renin-angiotensin system [56] that plays a critical role in the regulation of blood pressure and electrolyte and plasma volume homeostasis. Therefore, normal serum vitamin D levels help prevent hypertension through suppression of the renin-angiotensin system. In addition to the effect of vitamin D on the renin-angiotensin system, vitamin D can influence blood pressure through the suppression of vascular smooth muscle cell proliferation. It can also ameliorate insulin resistance, improve endothelial cell-dependent vasodilatation, and inhibit anticoagulant activity [57]. Vitamin D may modulate macrophage activity and cytokine production. Compared with uncomplicated pregnancies, preeclampsia is characterized by marked changes in vitamin D3 and calcium metabolism [58]. A recent meta-analysis and several observational studies show a significant relationship between vitamin D deficiency and an increased risk for preeclampsia [59–61]. Studies have shown that sufficient vitamin D intake during pregnancy reduces the risk of complications, including gestational diabetes, preterm birth, and infection [62, 63]. Maternal 25(OH)D3 levels are lower in women with preeclampsia than in normotensive pregnant women [64]. Moreover, a nested case control study revealed that maternal vitamin D deficiency at less than 22 weeks of gestation is a strong, independent risk factor for preeclampsia [65]. Placenta dysfunction plays an important role in the pathogenesis of this pregnancy disorder, since preeclampsia is associated with a reduced placental and fetal vitamin D pool [66]. Normal placental development and function ensure a healthy pregnancy outcome. It is believed that, during pregnancy, 1,25(OH)2D3 may be produced not only by kidneys but also by placenta trophoblasts. Human placenta and decidua are capable of producing and secreting 1,25(OH)2D3 [67]. The existence of gene transcript of 1*α*-hydroxylase and the finding of VDR expression in placental trophoblasts suggest a possible autocrine loop of vitamin D signaling within trophoblasts [68, 69]. A study by Zehnder et al. [70] also found that mRNA expression of 1*α*-hydroxylase was higher in the first- and second-trimester than in the third-trimester placentas, whereas mRNA expressions for VDR across gestation were less pronounced compared with 1*α*-hydroxylase. Though preeclampsia has been linked to maternal vitamin D insufficiency/deficiency [71, 72], the information on placental vitamin D metabolic system between normal and preeclamptic pregnancies is lacking. Preeclampsia is hypothesized to be a 2-stage disorder [73]. At its first stage, placental perfusion is reduced, often secondary to abnormal implantation. The poorly perfused placenta is proposed to produce materials that, in an appropriate maternal environment, initiate the ensuing multisystem sequelae (second stage). These pathophysiological changes are proposed to be secondary to abnormal endothelial function, which is a component of a generalized increase in the inflammatory activation [74]. The active form of vitamin D, 1,25(OH)2D, has been shown to regulate the transcription and function of genes associated with placental invasion, normal implantation, and angiogenesis [75]. Therefore, insufficient serum vitamin levels can impair normal functioning of these processes. Taken together, these findings about the association of maternal vitamin D deficiency and increased risk of preeclampsia emphasize the importance of adequate vitamin D levels and proper vitamin D metabolism during pregnancy.

## 5. Vitamin D, Infertility, and In Vitro Fertilization (IVF)

Accumulating evidence strongly indicates a potential role of vitamin D in human reproduction. Vitamin D receptors are present and differentially expressed in murine endometrium and ovary throughout the estrous cycle [76] whereas knock-out experiments have shown that vitamin D receptor null mice experience uterine hypoplasia and impaired folliculogenesis [77]. Moreover, a study in cell cultures confirmed the expression of vitamin D receptors in human endometrial cells and demonstrated that the expression of 1-alpha-hydroxylase, an enzyme which catalyzes the hydroxylation of calcidiol to calcitriol, is upregulated in the human endometrial stromal cells of early pregnant versus cycling endometria [78]. However, in vivo data supporting a role for vitamin D in female fertility in general and embryo implantation in particular are not robust. Finally, a recent retrospective study postulated that vitamin deficiency may negatively affect pregnancy rates with an effect mediated through the endometrium, given that vitamin D deficiency was not correlated with ovarian stimulation characteristics or with markers of embryo quality [79]. Up to date, only a few cohort studies have attempted to examine the role of vitamin D levels in infertile patients [79–82]. Results from these studies are strongly contradictory, with some findings showing that maternal vitamin D deficiency is associated with lower pregnancy rates [79, 81] and others demonstrating that vitamin D deficiency does not affect the final reproductive outcome [82, 83]. In addition, the number of patients enrolled in these studies is small, whereas the major shortcoming of all of the studies is the fact that no single embryo transfer policy has been adopted, with patients receiving up to four embryos per transfer. This indeed may severely bias the results, since it might be a strong confounding factor for estimating differences in pregnancy rates between vitamin deficient and replete women. A recent innovative study of Polyzos et al. [84] has evaluated the influence of vitamin D deficiency on pregnancy rates among women undergoing IVF/ICSI and Day 5 (blastocyst stage) single embryo transfer (SET). They have found that vitamin D deficiency results in significantly lower pregnancy rates in this setting of women. Overall 368 consecutive infertile women treated within a period of 15 months were included in the study. Clinical pregnancy rates were significantly lower in women with vitamin D deficiency compared with those with higher vitamin D values. Finally, even when restricting the analysis to women undergoing elective SET, vitamin deficiency was again independently associated with pregnancy rates. Vitamin D deficiency impairs pregnancy rates in women undergoing single blastocyst transfer. Future prospective confirmatory studies are needed to validate our results and examine the exact underlying mechanism by which vitamin D levels may impair pregnancy rates in infertile women undergoing IVF/ICSI.

## 6. Vitamin D and Endometriosis

Endometriosis, defined as the presence of endometrial glands and stroma in ectopic locations, affects 6%–10% of reproductive-age women. It is considered a chronic, estrogen-dependent, and inflammatory disease [85] associated with dysmenorrhea, dyspareunia, chronic pelvic pain, irregular uterine bleeding, and/or infertility [86, 87]. Actually, a unifying theory regarding the origin of endometriosis has remained elusive. Accumulating evidence is suggesting that dysregulation of Wnt and/or Hox genes may affect cell migration during organogenesis and differentiation of Müllerian structures of the female reproductive tract, with possible dislocation and dissemination of primordial endometrial stem cells in ectopic regions, which have high plasticity to differentiation [88]. It is possible that, during postpubertal age, under the influence of different stimuli, these misplaced and quiescent ectopic endometrial cells could acquire new phenotype, biological functions, and immunogenicity. So, these kinds of cells may differentiate, specializing in epithelium, glands, and stroma, to form a functional ectopic endometrial tissue. This may provoke a breakdown in the peritoneal cavity homeostasis, with the consequent processes of immune alteration, documented by peripheral mononuclear cells recruitment and secretion of inflammatory cytokines in early phases and of angiogenic and fibrogenic cytokines in the late stages of the disease [89]. An association has been postulated between endometriosis and vitamin D, since endometriosis is a disease that mimics malignancy and fulfills most of the criteria of an autoimmune disease, and vitamin D is an agent with antiproliferative, anti-inflammatory, and immunomodulatory properties [90–92]. Endometriosis has many features of an autoimmune disease, and an immune-mediated defect in recognition and elimination of endometrial fragments refluxed in the peritoneal cavity has been proposed to play a crucial role in endometriosis development [93]. Activated CD4^+^CD8^+^ lymphocytes, macrophages, and dendritic cells express widely VDR and both the activating and metabolizing enzymes, 1-*α* hydroxylase and 24-hydroxylase [94, 95]. This suggests that 1,25(OH)2D can be produced locally in the immune system and plays an autocrine-paracrine role [96]. However the link between endometriosis as an autoimmune disease and vitamin D as an immunomodulator is more complex. In fact, endometriosis is associated with normal or high 25(OH)D reserve, rather than insufficiency/or deficiency, as would have been expected. In addition, the manifestations of endometriosis do not exhibit seasonal flares or exacerbations or seasonal changes in 25(OH)D levels as in other autoimmune diseases. It is plausible that the immunomodulatory role of vitamin D in this disease, if existent, is local, autocrine, and/or paracrine, at the level of endometriotic foci or lesions. If so, it would be missed by correlating disease manifestations with circulating serum 25(OH)D levels and could only be identified by targeted in vitro studies, which to the best of our knowledge are lacking [97]. In a recent large prospective cohort study, a greater predicted plasma 25(OH)D level was associated with a lower risk of endometriosis [98]. However, other studies have failed to demonstrate this association [90, 99]. This could be explained by the small sample size, heterogeneity, and case-control nature of these previous studies. Finally, recently, a randomized double-blind study was conducted in order to evaluate the role of vitamin D in primary dysmenorrhea: women received vitamin D supplementation before their expected menses (*n* = 20) or placebo (*n* = 20); a significant reduction (41%) in the mean pain score was noticed in the experimental arm [100]. The greatest reduction of pain scores was observed in the subset of more symptomatic patients at baseline. The pain reduction could be attributed to the action of 1,25(OH)2D on the endometrium with a decrease in prostaglandin synthesis and an increase in prostaglandin inactivation by suppression of cyclooxygenase 2 and upregulation of 15-hydroxyprostaglandin dehydrogenase, respectively. 1,25(OH)2D may also exert anti-inflammatory effects through other pathways, such as inhibiting nuclear factor-*β* signaling and increasing mitogen-activated protein kinase phosphatase 5 activity, thus blocking cytokine production via p38 activation [101]. Actually, the medical treatment of endometriosis is not satisfactory, and there is a constant need to find novel drugs with better efficacy and tolerability. These findings show the role of vitamin D as a possible modifiable risk factor for endometriosis. Use of vitamin D supplementation in these patients, especially when exhibiting low plasmatic levels of 25(OH)D, may allow these women to limit the use of nonsteroidal anti-inflammatory drugs [100]. However, larger, placebo-controlled studies are needed to clarify the possible favorable effects of vitamin D supplementation in women with endometriosis.

## 7. Vitamin D and Breast and Ovarian Cancer

Biological and epidemiological data have revealed the protective functions of vitamin D against ovarian, breast, colorectal, gastric, liver, prostate, and nonmelanoma skin cancers [102–104] and the potential role of VDR gene polymorphisms and risk of cancer [105–107]. The most frequently studied single-nucleotide polymorphisms are the restriction fragment length polymorphisms FokI (rs2228570) and BsmI (rs1544410), as defined by the endonucleases FokI and BsmI [105]. As widely reviewed by Vuolo et al. [91], several levels of evidence support the relationship between vitamin D and cancer: firstly, low circulating levels of vitamin D are associated with increased risk of developing cancer, secondly a high intake of vitamin D is associated with a reduced risk of cancer, in addition the aggressiveness of cancer is lower in summer when the production of vitamin D is higher, and finally polymorphisms of genes encoding proteins involved in the signal pathway of vitamin D affect the risk of developing cancer. However, data about vitamin D and cancer are often conflicting, with a considerable variability [106–109]. Upregulation of VDR expression has been shown in several tumors and is thought to represent an important endogenous response to tumor progression [106, 107].

Of particular interest in this regard, it is estimated that 20% of all cancer cases are caused by obesity in women [110] and vitamin D is thought to be one of the mechanisms underlying this association. Shanmugalingam et al. [111] performed a systematic review and meta-analysis in order to assess whether vitamin D plays a role in the pathway between obesity and cancer, since no mediation analyses have been performed for this effect to date. For the topic obesity and cancer, a positive association was reported between obesity and risk of cancer, showing that the strength of this association varies between cancer sites, sex, and, in breast cancer, the menopausal status. There are several molecular mechanisms suggested to explain the increased risk of cancer in obese people: firstly, the “insulin-cancer hypothesis” [112]; secondly, in hormonally driven cancers, such as endometrial and postmenopausal breast cancer, the increase in circulating levels of sex steroid hormones. In the postmenopausal state, the majority of oestrogen is derived from adipose tissue rather than from the ovaries, potentially explaining the discrepancy between pre- and postmenopausal women. Finally, obesity is thought to result in a state of chronic inflammation. These changes lead to an increase in tumor cell motility, invasion, and metastasis. For the link between obesity and vitamin D, the meta-analysis reported a modest inverse association between obesity and low vitamin D levels. The underlying biological mechanisms are still unknown. The most likely hypothesis is that vitamin D stored in fat tissue increases local vitamin D concentrations causing activation of the VDR in adipocytes. This may lead to low energy usage and further promotion of obesity [111, 113]. Finally, authors evaluated if vitamin D is a mediator for the association between obesity and cancer. Even if the literature shows consistent evidence for an association between vitamin D and obesity, there was lack of studies showing a consistent link between vitamin D and cancer after adjustment for obesity. Authors concluded that it seems that the significance of the mediating role of vitamin D in the biological pathways linking obesity and cancer is low [111].

Breast cancer is one of the most common female cancers in the Western population and there is a growing interest in identifying the role and the relative importance of environmental risk factors, lifestyle, and diet in this type of tumor. There are laboratory data that support the hypothesis that the anticarcinogenic effects of vitamin D could be mediated via the oestrogen pathway by downregulation of the oestrogen receptor (ER) and thus attenuating oestrogenic bioresponses such as cell growth [114, 115]. Also for breast cancer, VDR has a crucial role. Both healthy and cancer breast cells express the VDR and gene ablation studies have shown a role of VDR in physiological mammary gland development. Zinser and Welsh [116] showed that, after the stimulation with the carcinogen DMBA, mice knockout for VDR gene developed a higher number of premalignant lesions compared to wild-type mice. The antiproliferative and prodifferentiating effects of vitamin D seem to regulate differentiation in the breast by a balance between the activity of the 1*α*-hydroxylase and 24-hydroxylase enzymes, responsible, respectively, for the synthesis and degradation of the active hormone 1,25(OH)2D. Several studies have found an increased expression of CYP24 in tumor cells compared to healthy cells, suggesting that the malignant tissue present a tendency to the degradation of active vitamin D [117]. Recently, Narvaez et al. [118] reviewed the frequency of genomic VDR changes in human breast cancers using data sets available on The Cancer Genome Atlas. Alterations in the VDR gene were present in only 5% of human breast tumors. The most common change was a reduction in mRNA expression. Intriguingly, the Luminal B subtype had the highest frequency of VDR alterations with 10.5% of tumors displaying reduced VDR mRNA expression compared to 0–3% for Luminal A, Basal, HER2, or Claudin-Low subtypes. These results are in line with the data of Santagata et al. [119]; interestingly, this study also demonstrated that breast tumors with the highest expression of VDR, ER, and androgen receptor had the best prognosis. Several prospective epidemiological studies have investigated the relationship between vitamin D and breast cancer incidence, but with conflicting results [103, 120–122]. Recently, a meta-analysis of prospective studies reported that a 12,5 nmol/L (5 ng/mL) increase in 25(OH)D was linked to lower risk of postmenopausal (but not premenopausal) breast cancer with RR of 0.88 per 12,5 nmol/L (5 ng/mL) (95% CI, 0.79 to 0.97) [123]. However, Amir et al. [124] did not find a significant association of 25(OH)D levels with breast cancer risk. Stronger evidence is available from four randomized trials that have evaluated the effect of vitamin D supplementation on risk of bone fracture and mortality; they examined risk of cancer as a secondary outcome [125–128]. The United Kingdom trial compared vitamin D supplementation to placebo; no cancer risk reduction was observed [125]. In the Nebraska trial, patients were randomly assigned to receive calcium and vitamin D, calcium alone, or placebo; there was no direct comparison of vitamin D alone versus placebo [126, 127]. There were few cancers overall; however, those randomized to calcium/vitamin D supplementation (versus placebo) had a lower overall cancer incidence. The larger Women's Health Initiative trial did not show a reduction in risk of colorectal, breast, or any cancer in those randomized to vitamin D [127]. Finally, in the Record trial, vitamin D versus placebo administration was not associated with a reduction in cancer risk [128]. These largely negative results may reflect inadequate vitamin D dosing, small sample sizes, or the true absence of an association. The promising findings of the Nebraska study may reflect the combined use of calcium and vitamin D, rather than vitamin D alone. Regarding the cancer outcome, a recent meta-analysis of 42 RCTs reported an inverse correlation between vitamin D supplementation and all-cause mortality; cancer specific mortality was not examined [129]. For established breast cancer, a meta-analysis of eight studies has found that low vitamin D levels were linked to higher risk of recurrence and death; however, these results may be flawed by many bias and confounding factors in the included studies [130]. Three of the RCTs discussed above (United Kingdom, WHI, and Record) also evaluated cancer outcomes. No significant difference in cancer death between the vitamin D and non-vitamin D supplementation arms was identified in these three studies (HR 0.86, 95% CI, 0.61 to 1.20; HR 0.90, 95% CI, 0.77 to 1.05; and HR 0.85, 95% CI, 0.68 to 1.06, resp.) [125, 127, 128].

Ovarian cancer is one of the most lethal gynaecological malignancies, with an estimated 225,500 new cases and 140,200 deaths worldwide annually [131]. Epidemiological and laboratory studies have shown that the vitamin D endocrine system may be involved in ovarian carcinogenesis. The presence of VDR in normal ovarian epithelium, in human ovarian tumors, and in human ovarian cancer cell lines has been demonstrated [10]. For the ovarian cancer cell line, OVCAR-3, 1a,25(OH)2D reduces the proliferation induced by dihydrotestosterone through the VDR [132]. In ovarian cancer cells, 1a,25(OH)2D leads to G2/M cell cycle arrest through a p53-independent induction of GADD45, which modulates tumor formation [133]. VDR is necessary for full ovarian function through direct effects on oestrogen biosynthesis and regulation of aromatase gene expression [134]. Additionally, it may also antagonize androgen, which has been suggested to play an important role in ovarian carcinogenesis [135, 136] by inhibiting the androgen receptor expression which was found in the majority of ovarian tumors [132]. Interestingly, VDR has been found to be upregulated in ovarian tumors when compared with nonmatched normal ovarian tissue [137]. With regard to ethnical differences, substantial racial variation has been observed in the incidence of ovarian cancer, with highest rates in Caucasian women and lowest rates among Asian women [138]. This fact might be partially explained by differences in variant allele frequencies, the association of VDR polymorphisms with ovarian cancer risk being generally inconsistent among ethnic groups [134].

## 8. Conclusions

A large body of recent evidence suggests that abnormalities of vitamin D levels and its signaling may play a key role in the development of gynecological/obstetric pathologies in various age periods of woman's life, including selected oncological diseases. VDR-mediated signaling pathways and vitamin D levels seem to (significantly) affect the risk of several gynecological diseases, such as PCOS, endometriosis, and ovarian and even breast cancer. Moreover, since also the maternal-fetal unit is under the influence of vitamin D, a breakdown in its homeostasis may underlie infertility, preeclampsia, and GDM. According to our literature review, the relationship between vitamin D and gynaecological/obstetric diseases must be replicated in future studies which could clarify the molecular machineries behind their development. We suggest that further investigation should take into account the different serum levels of this vitamin, the several actions which arise from the binding between it and its receptor (taking into account its possible polymorphism), and finally the interplay between vitamin D metabolism and other hormonal and metabolic pathways.
